# Assisted dispersal and reproductive success in an ant species with matchmaking

**DOI:** 10.1002/ece3.9236

**Published:** 2022-08-23

**Authors:** Mathilde Vidal, Jürgen Heinze

**Affiliations:** ^1^ LS Zoologie/Evolutionsbiologie Universität Regensburg Regensburg Germany

**Keywords:** Formicidae, Hymenoptera, mating behavior, outbreeding, polyandry, population structure

## Abstract

Workers of the ant *Cardiocondyla elegans* drop female sexuals into the nest entrance of other colonies to promote outbreeding with unrelated, wingless males. Corroborating the results from previous years, we document that carrier and carried female sexuals are typically related and that the transfer initially occurs mostly from their joint natal colonies to unrelated colonies. Female sexuals mate multiply with up to seven genetically distinguishable males. Contrary to our expectation, the colony growth rate of multiple‐mated and outbred female sexuals was lower than that of inbred or single‐mated females, leading to the question of why female sexuals mate multiply at all. Despite the obvious costs, multiple mating might be a way for female sexuals to “pay rent” for hibernation in an alien nest. We argue that in addition to evade inbreeding depression from regular sibling mating over many generations, assisted dispersal might also be a strategy for minimizing the risk of losing all reproductive investment when nests are flooded in winter.

## Graphical abstract

The paternity analyses of workers produced by transported female sexuals in a matchmaking ant species show that females can mate multiply. However, if polyandry can be beneficial, it appears, here, to be correlated with a decrease in colony growth rate. This study proposes evolutionary explanations for the persistence of polyandry in *Cardiocondyla elegans*.

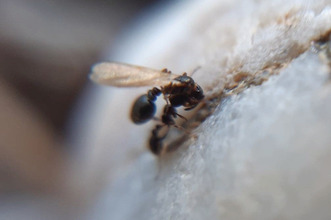

## 1 INTRODUCTION

In many animals, offspring dispersal is critical to avoid local resource competition and the risk of inbreeding. Given that dispersal is often dangerous and young individuals have limited knowledge about the specific conditions in their surroundings, parents may assist their young during dispersal. For example, poison frogs may transfer tadpoles from land to water (Pašukonis et al., 2019), wolf spiders may carry spiderlings (Bonte et al., 2007), and mother bats teach their offspring to navigate and find suitable habitats (Goldshtein et al., 2022). Similarly, in several species of ants, workers accompany and even carry young queens during the founding of new colonies away from their natal nests (e.g., Fernández‐Escudero et al., 2001; Möglich & Hölldobler, 1974; Peeters & Aron, 2017).

**FIGURE 1:**
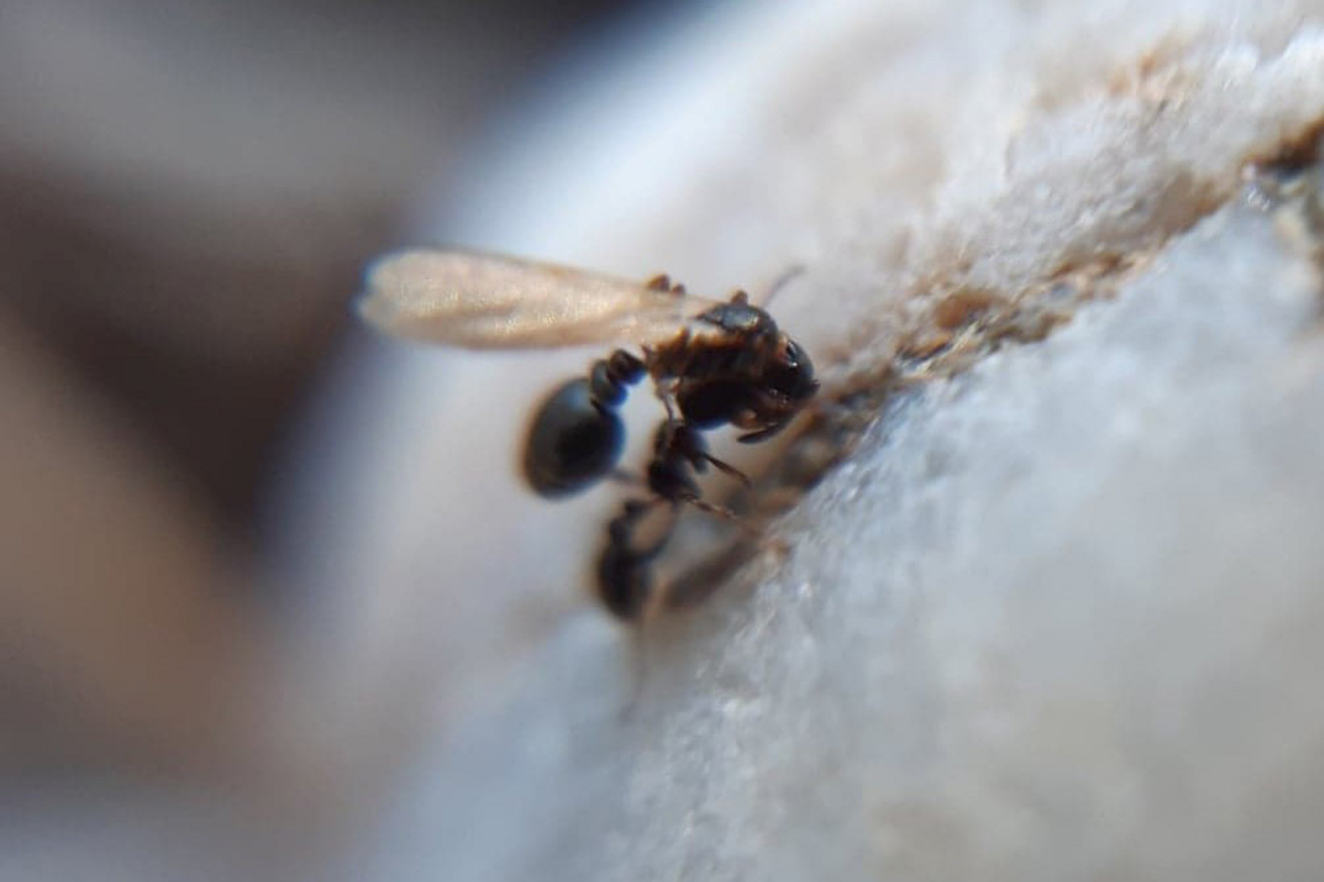
Picture of a worker carrying a winged female sexual in the ant *Cardiocondyla elegans* to the nest entrance of another colony (Beaucaire, Gard, France, 2020; photo by M. Vidal).

In the ant *Cardiocondyla elegans*, workers may carry their female sexual sisters over several meters and dump them into the nest entrance of another colony. We have argued that this peculiar behavior constitutes a type of assisted dispersal and mate choice, allowing young queens to outbreed with several unrelated males away from their natal nests (Vidal et al., 2021). The ant genus *Cardiocondyla* is characterized by an ancestral male polyphenism with winged disperser males and wingless “ergatoid” males, which locally mate with female sexuals eclosing in their natal nests. Colonies of most tropical *Cardiocondyla* are relatively small, with only a few dozen workers and one or several queens. Individual wingless males are therefore capable of monopolizing mating with all female sexuals by aggressively excluding other males with their shear‐ or sickle‐shaped mandibles. In contrast, colonies of Palearctic species are typically much larger, with several hundred workers and obligatorily a single, multiply mated queen. Furthermore, female sexuals are produced not year‐round as in the tropics but seasonally. Because of this, wingless males are no longer capable of individually securing copulations with all female sexuals and indeed have evolved mutual tolerance (Heinze, 2017). As mating occurs in the natal nest, inbreeding coefficients determined from microsatellite genotypes are relatively high. Nevertheless, data suggest that 40%–80% of matings involve unrelated sexuals (Lenoir et al., 2007; Schrempf, 2014; Schrempf, Heinze, et al., 2005; Schrempf, Reber, et al., 2005; Vidal et al., 2021), and observations in the field revealed that in *C. elegans* these are facilitated by “royal matchmaking” by the workers, which transfer female sexuals between unrelated nests (Figure 1). Mating frequencies suggest that female sexuals mate multiply with both related and unrelated males and presumably are carried repeatedly among nests before they finally hibernate in an unrelated nest. Aggression among female sexuals has not been observed, but as colonies of *C. elegans* obligatorily contain only a single egg‐laying queen, they leave after hibernation, disperse on foot or, when still bearing wings, by drifting with the wind and presumably found their own nests solitarily (Lenoir et al., 2007; Vidal et al., 2021).

Here, we follow up on our earlier studies and provide new data about queen carrying in the field, the relatedness among carrier and carried ants, and the growth rate of colonies started by individual queens after mating only with brothers, one or multiple unrelated males. We expected that outbreeding and multiple mating are reflected in a better performance of colonies, that is, faster colony growth.

## 2 MATERIAL AND METHODS

### 2.1 Study species and collection

*Cardiocondyla elegans* Emery 1869 is a thermophilic ant that builds its nests in the soil in barely vegetated, sandy patches, for example, along rivers in Iberia, France, and Italy. Populations in the Eastern Mediterranean are now considered to represent a separate species, *C. dalmatica* Soudek 1925 (Seifert, 2018). From 2014 to 2017, we studied several populations of *C. elegans* along the rivers Gardon and Rhone in Southern France. In 2018 and 2019, we concentrated our study on one site south of Remoulins on Gardon (N 43°55′43.9″, E 4°34′5.1″), where we repeated our investigation in August 2020 to corroborate earlier results and retrieve additional detail about the peculiar phenomenon. For genetic analyses, we also used samples collected at the same site in August 2019.

The locations of nests were identified by following foragers back to the nest entrance. Samples of workers were stored in EtOH for the determination of nestmate relatedness by genotyping the individuals. By observing nest entrances and following individual pairs of carrying workers and carried female sexuals, over several minutes, we attempted to identify the direction of the transfer. For genetic analyses, we collected the ants with an aspirator immediately before the female sexual was placed into the entrance of the recipient colony. In addition, we collected pairs of unknown origin and destination and either stored them in 100% EtOH for the determination of genetic relatedness or kept them alive for later experiments.

### 2.2 Genetic analyses

We determined the genotypes of workers and female sexuals at seven microsatellite loci as described before (CE2‐3A, CE2‐4A, CE2‐4E, CE2‐5D, CE2‐12D, Card 8, and Cobs 13; e.g., Lenoir et al., 2007; Vidal et al., 2021). In short, DNA was extracted from whole ants using the CTAB method (modified from Sambrook & Russel, 2001) and diluted in 30 μl of TE buffer. Polymerase chain reactions (PCRs) were performed in a 20 μl reaction volume using 1 μl of DNA with 19 μl of master‐mix (7 μl H2O, 10 μl GoTaq, and 1 μl of each forward and reverse primers). Samples were amplified following Lenoir et al. (2005) with an initial denaturation step at 94°C for 3 min, 40 cycles at 94°C for 45 s, with an annealing temperature according to the primer for 45 s (Table S1), followed by a step of 72°C for 45 s, and a final extension step at 72°C for 7 min. Allele sizes were determined by GeneScan® 3.1 software (Applied Biosystems) and GeneScan® 500 TAMRA dye as a size standard. Relatedness among nestmates and pairwise relatedness between female sexuals and their carriers were calculated from these genotypes following Queller and Goodnight (1989) using the *related* package (Pew et al., 2015) on R software‐4.0.3 and Relatedness v4.2 (Queller & Goodnight, 1994). Fixation coefficients (*F*_ST_) were estimated using GenAlEx v6.51b2 (Peakall & Smouse, 2006). The frequency of sibling mating *α* was calculated from *F*_ST_ = *α*/(4−3*α*) (Pamilo, 1985).

### 2.3 Colony maintenance

**FIGURE 2:**
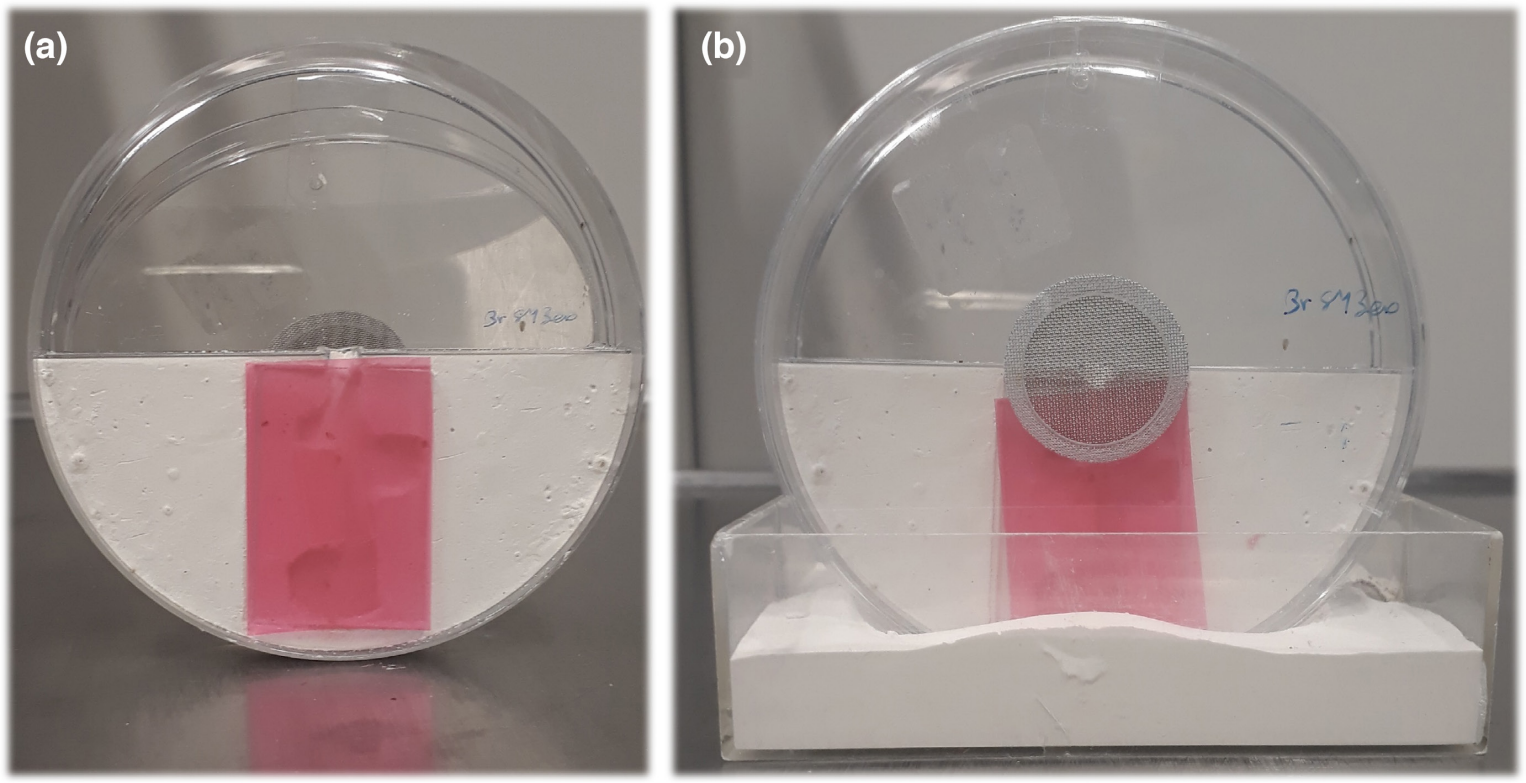
Small artificial vertical nests for the maintenance of *Cardiocondyla elegans* under near‐natural conditions in the laboratory. Both pictures show a plaster nest, either open (a) or closed and with a plaster basis to keep it in an upright position (b). Isolated queens were kept in these nests, and their offspring production was monitored by opening the lid and through the transparent red plastic (photo by M. Vidal).

Individual life pairs of a carrying worker and a carried female sexual were kept in 1.5‐ml Eppendorf tubes with moist cotton and fed with cookie crumbs. After transfer to the laboratory, we added to each of 43 female sexuals one to three unrelated males from laboratory stock colonies (one male to 16, two males to 13, and three males to 14 female sexuals). Additional 112 female sexuals were left without the chance to mate with additional males. One day later, the males were removed and the female sexuals were individually placed into 94 mm × 16 mm Petri dishes divided by a separator into two equal halves. One half of the dish was filled with plaster with small tunnels and two or three small chambers serving as the nest, the other served as a foraging arena, into which we added a droplet of honey, dead *Drosophila* or chopped up cockroaches twice per week. To allow the ants to move from the nest to the foraging arena, we made a 5 mm wide hole in the plastic separator using a soldering iron. The nesting chambers were covered by red plastic foil. To more closely mimic the architecture of natural nests, the Petri dishes were kept vertically by attaching them to a plaster base (Figure 2). Experimental nests were kept in incubators under near‐natural artificial conditions matching those found at the collecting site (i.e., from June to September at summer temperatures of 17–34°C, slowly changing by a weekly temperature decrease of up to 4°C to 5–13°C in December to March). Offspring production of isolated female sexuals was recorded every week for a total of 280 days. In addition, we observed offspring production from similarly housed laboratory‐reared female sexuals, which had had only access to brothers. These latter colonies were kept alive only for 84 days after the hibernation. Note here that we use “female sexuals” even for individuals that have mated and began laying eggs and which would conventionally be referred to as “queens.” However, as many data refer to both mated, fertile and virgin, nonfertile individuals, using both terms would have made the text difficult to read.

In addition to the population genetics software mentioned above, statistical analyses were made with R 4.0.3 (Frasier, 2020) and Statistica 6.0 (StatSoft).

## 3 RESULTS

### 3.1 Population and colony genetic analyses

Nestmate relatedness and inbreeding coefficients were determined from 42 workers from six colonies collected in 2019 and 113 workers from 16 colonies collected in 2020. Mean relatedness per colony ranged from 0.18 to 0.66 in 2019 and 0.13 to 0.69 in 2020. Across all studied colonies, the mean relatedness was 0.49 ± SE 0.08 in 2019 and 0.39 ± 0.04 in 2020, which is in the range of earlier estimates (0.34 ± 0.05, Vidal et al., 2021). The same applies to the fixation coefficient (2019: 0.43 ± 0.10, 2020: 0.28 ± 0.03, 2018: 0.30 ± 0.03).

As colonies are obligatorily monogynous, that is, have only one fertile queen, the lower relatedness suggests multiple mating, which is also evident from the number of genotypes found among nestmates. The mean effective mating frequency of queens calculated from nestmate relatedness following Tarpy and Nielsen (2002) is 2 and 3.5 in our samples from 2019 to 2020, respectively. The actual mating frequencies of individual queens that had been collected when carried in the field, estimated from the genotypes of offspring reared in the laboratory, revealed a large variation from one to seven mating partners per queen (mean 4.3 ± SD 1.6, *n* = 6). Although adding unrelated males to female sexuals in the laboratory resulted in additional mating as reflected in worker genotypes, mean mating frequencies did not differ (mean 3.3 ± 2.3, *n* = 12; *t*‐test, *t* = 0.950, *p* = .346).

The relatedness between carrier and carried female sexual was 0.39 ± 0.03 in 2019 (*n* = 91) and 0.23 ± 0.08 in 2020 (*n* = 8), that is, in the range of colony‐wise estimates for relatedness but, in contrast to our earlier study (Vidal et al., 2021), significantly lower than the estimated mean relatedness in these years (*t*‐test, 2019: *t* = 2.90, *p* = .005; 2020: *t* = 2.86, *p* = .009). The relatedness of female sexuals to source colonies was 0.29 ± 0.14 (*n* = 3) in 2019 and 0.21 ± 0.06 (*n* = 8) in 2020, and the respective values for recipient colonies were 0.03 ± 0.21 and −0.04 ± 0.07. Relatedness to the source colony was not different from nestmate relatedness in 2019 (*t* = 1.98, *p* = .09) but significantly lower in 2020 (*t* = 3.81, *p* = .001). Relatedness to the target colony did not differ from zero (single‐sample *t*‐test, 2019: *t* = 0.25, *p* = .83; 2020: *t* = 1.62, *p* = .15).

The large number of pairs of carriers and female sexuals collected in 2019 allowed us to estimate how pairwise relatedness varied with time. The probability of carrier and female sexual being less closely related than 0.25 (the value for halfsisters) increased from 0.17 in the first phase of our field collection (Aug 14–16, *n* = 6) to 0.43 in the seventh and last phase (Aug 30, *n* = 14; Spearman rank correlation, *r*_S_ = .81, *p* = .035).

### 3.2 Colony growth rate and female mating frequency

**FIGURE 3:**
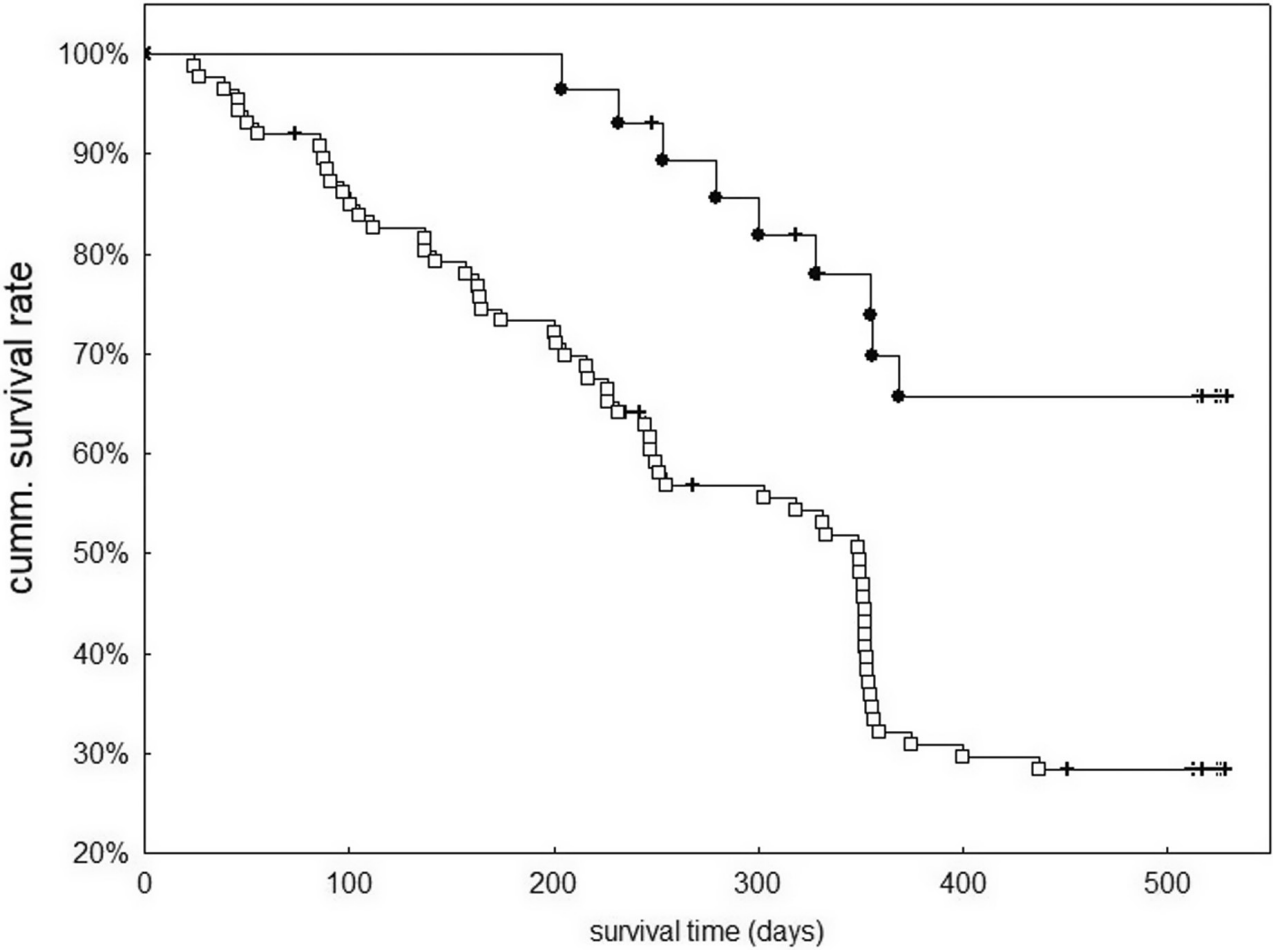
Survival time of female sexuals of the ant *Cardiocondyla elegans* from Southern France that produced worker offspring in the laboratory (○) or failed to do so (￭). The symbol + indicates censored information. Unproductive female sexuals had a significantly shorter life expectancy.

A considerable percentage of female sexuals, which had been transferred to the laboratory and kept in isolation, died within the first 28 days of the experiment (40% of 43 female sexuals with and 26% of 112 female sexuals without temporary access to males in the laboratory). Of the surviving individuals with access to males, 23 (88%) produced at least one worker compared with 13 (16%) of those without. Female sexuals, which succeeded in producing offspring over the 530 days of the experiment (*n* = 36), lived significantly longer than those that did not (*n* = 80; log‐rank test, test statistic = −7.57, *p* < .0001, Figure 3). Dissection of four female sexuals that died or did not produce any workers revealed an empty spermatheca, suggesting that also other unproductive female sexuals had been unmated.

**FIGURE 4:**
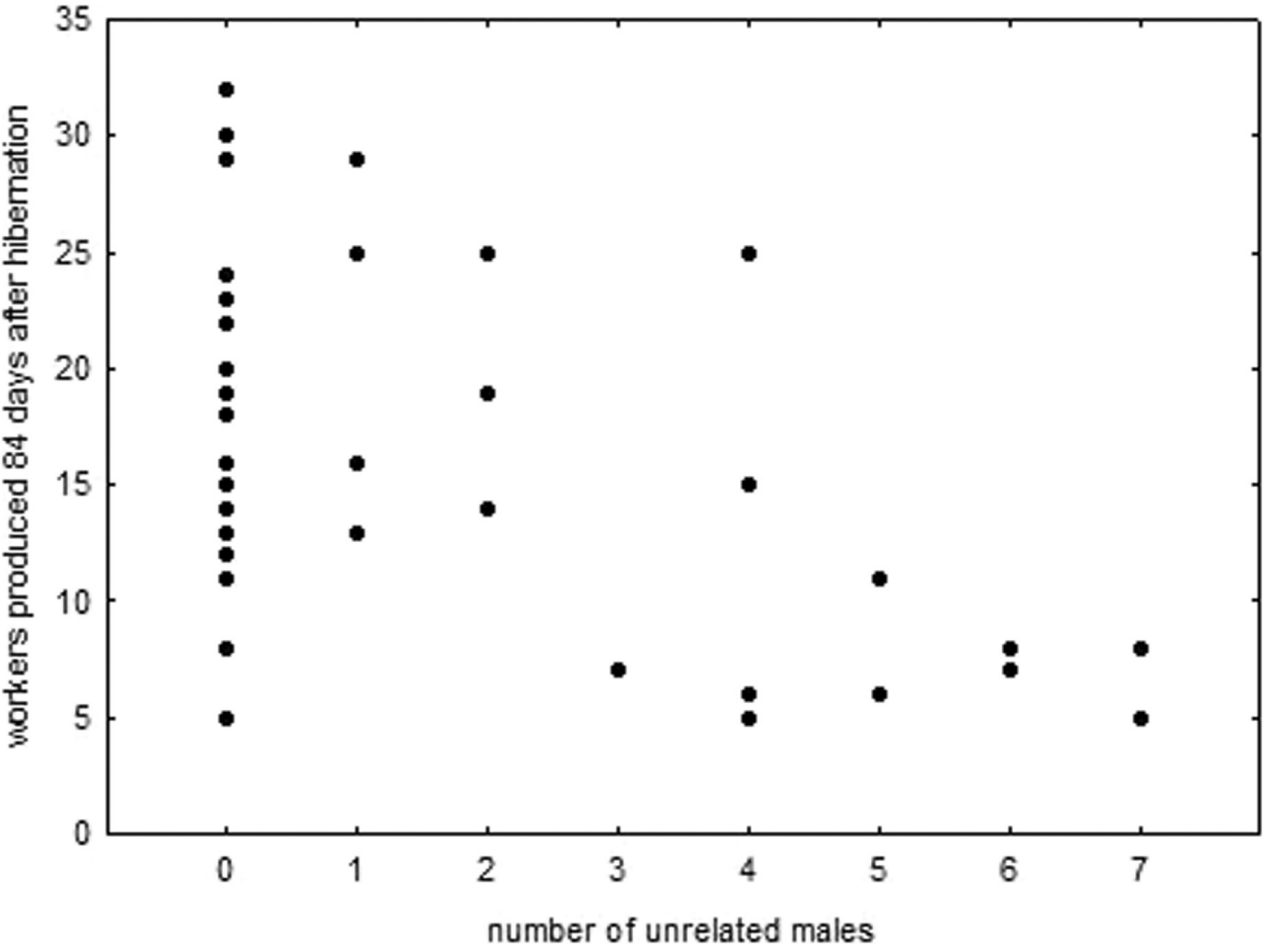
Offspring production by female sexuals of the ant *Cardiocondyla elegans* that had access only to brothers or in addition to one to seven unrelated males. Multiple‐mated individuals appeared to produce considerably fewer offspring than inbred or outbred, single‐ or double‐mated female sexuals.

The number of offspring produced by queens in the laboratory with or without access to males varied significantly with mating frequency as determined by paternity analysis (Kruskal–Wallis test, *H*_[6,18]_ = 14.97, *p* = .02). Many queens had already mated in the field and not all mated with the additionally added males in the laboratory. Surprisingly, average productivity declined with mating frequency (Spearman rank correlation, *r*_S_ = −.816, *p* < .001, Figure 4). Furthermore, a comparison between these field‐collected and laboratory‐reared female sexuals, which could only have mated with their brothers, did not reveal a difference in the number of offspring reared during the first 84 days after hibernation (Kruskal–Wallis test, *H*_[19,40]_ = 20.80, *p* = .348, Figure 4).

## 4 DISCUSSION

Our new population and colony genetic data corroborate earlier estimates (Vidal et al., 2021) that colonies of the ant *C. elegans* in Southern France typically harbor a single, multiply mated queen and that a large percentage of mating episodes involve siblings. Workers promote outbreeding by commonly transferring female sexuals between nests. We have previously hypothesized that during the mating period in summer, female sexuals are carried repeatedly from nest to nest (Vidal et al., 2021). While first related workers will carry them away from their natal nests, later unrelated workers will move them from a first unrelated recipient colony to another. Our data appear to support this hypothesis. First, the percentage of carriers and female sexuals that were less closely related than 0.25 increased from mid‐ to late‐August 2019. Second, in contrast to our previous study (Vidal et al., 2021), the average relatedness between carrier and carried female sexual was significantly lower than mean nestmate relatedness in both years, probably because in 2019 and 2020, more sexuals were collected late in the mating season. Third, genotyping the offspring of carried female sexuals showed that several of them had mated with six or seven genetically distinguishable males, which suggests the “visit” of more than one unrelated colony.

A large fraction of field‐collected female sexuals died without producing workers when housed in artificial nests in the laboratory. Dissections of a small number of corpses, in which the ovaries had not yet decayed, revealed the absence of sperm in the spermatheca, suggesting that also other unproductive individuals had been virgins. Their higher mortality matches the finding that in social insects mating and reproduction increase life expectancy (e.g., Hartmann & Heinze, 2003; Schrempf, Heinze, et al., 2005; Schrempf, Reber, et al., 2005), in contrast to many solitary insects, in which virgins outlive reproductively active, mated females (e.g., Chapman et al., 1998; Limberger et al., 2021; Service, 1989). A positive association between fecundity and longevity appears to be a trait associated with the evolution of perennial insect societies (e.g., Jaimes‐Niño et al., 2022; Parker, 2010; Rodrigues & Flatt, 2016).

Contrary to our expectation, colony growth rates were highest in colonies with a singly or doubly mated queen, while colonies with queens with higher mating frequencies were much less productive. Multiple mating has been shown to increase colony growth rate in the western harvester ant (*Pogonomyrmex* occidentalis; Wiernasz et al., 2004), increase the resistance to pathogens in bees and wasps (e.g., Saga et al., 2020; Seeley & Tarpy, 2006; Shykoff & Schmid‐Hempel, 1991), and a more efficient division of labor (e.g., Oldroyd & Fewell, 2007). As in our study, colony growth rate was monitored only for about 1 year and no sexuals were produced during this time, we cannot exclude that multiple mating in *C. elegans* might have a beneficial effect later in the life of a colony or under more variable environmental conditions in the field.

Laboratory‐reared queens that were only given the chance to mate with their brothers produced similar numbers of offspring during the first 84 days after hibernation as single‐ or double‐mated queens. This means that outbreeding does not immediately lead to a higher fitness than a single round of inbreeding. In honeybees and many other Hymenoptera with single locus complementary sex determination (sl‐CSD), inbreeding results in the production of sterile or inviable diploid males (e.g., Darvill et al., 2012; Van Wilgenburg et al., 2006). While *Cardiocondyla* appears to have a different mechanism of sex determination, in which sister–brother and even mother–son mating does not result in the production of diploid males (Schmidt et al., 2013; Schrempf et al., 2006), repeated sib‐mating nevertheless results in decreased life span and reproductive success (Schrempf et al., 2006). That a single round of sib‐mating does not immediately result in inbreeding depression in *C. elegans* does therefore not exclude any possible negative effects of inbreeding over several generations.

The significant initial costs of multiple mating and the efforts associated with assisted outbreeding raise the question of why *C. elegans* has evolved the carrying of female sexuals and polyandry. Female sexuals may mate “for convenience” when resistance to courting males is too costly, as has been suggested for the polyandrous queens of *Plagiolepis pygmaea* (Trontti et al., 2007). However, previous observations of mating behavior in *Cardiocondyla* clearly show that female sexuals are well capable of defending themselves even against the most vicious courtship by much larger males (e.g., Heinze et al., 2021). Instead, female sexuals of *C. elegans* might copulate repeatedly “to pay rent” for hibernation in an alien colony. Colonies should not tolerate alien queens, which already mated with alien males and are no longer available for mating with the workers' brothers, as this would pose the risk of usurpation and queen replacement without fitness benefits. If the adopted queen mated with the resident males, this might increase the potential reproductive success of the latter and therefore the inclusive fitness of the workers. Such a hypothesis might also explain why workers carry alien female sexuals from their nest to other, unrelated recipient colonies—doing so, they disperse the sperm of their brothers without risking that their own queen is later replaced, in a way passing the buck like in the “Black Peter” or “Old Maid” card game, in which players try to win by getting rid of an unmatchable card. In any case, by exporting female sexuals to other nests, colonies increase the likelihood that their sexual offspring survive hibernation. Temporary flooding of nests on the banks of Loire during winter results in high colony mortality (Lenoir et al., 2007) and assisted dispersal may be a reasonable risk‐averse strategy in unpredictable environments.

## AUTHOR CONTRIBUTIONS

**Mathilde Vidal:** Data curation (lead); investigation (lead); writing – review and editing (supporting). **Jürgen Heinze:** Conceptualization (lead); data curation (supporting); investigation (supporting); writing – original draft (lead).

## FUNDING INFORMATION

Open Access funding enabled and organized by Projekt DEAL.

## CONFLICT OF INTEREST

None declared.

## Supporting information


Table S1

## Data Availability

Raw data, individual genotypes, pairwise relatedness estimates, and colony growth data are available at https://doi.org/10.6084/m9.figshare.19982081.
