# Supplementary material for: Assisted dispersal and reproductive success in an ant species with matchmaking
Source: Ecol Evol. 2022 Aug 23;12(8):e9236. doi: 10.1002/ece3.9236 (PMC9398888; doi:10.1002/ece3.9236)
Supplement: Supplementary file 1 — Table S1 [file ECE3-12-e9236-s001.docx]

**Assisted dispersal and reproductive success in an ant species with matchmaking**

Mathilde Vidal ǀ Jürgen Heinze

LS Zoologie / Evolutionsbiologie, Universität Regensburg,
Universitätsstraße 31, 93040 Regensburg, Germany

**Table S1:** Primer microsatellites used for the ant *Cardiocondyla elegans* with: primer sequences, repeat motif, primer pair-specific annealing temperature (TA), size range in base pair, observed number of alleles, and fluorescent primer labels.

| Locus name | Primer sequences (5′to 3′) | Repeat type | TA   (°C) | Size range (bp) | No. of alleles | Label of F‐primer |
| --- | --- | --- | --- | --- | --- | --- |
| CE2–3A | F: CCGTCTTTTCCACTCAC R: GGAATCGTCGAGAGAGA | (AG) | 60 | 97–135 | 18 | TET |
| CE2–4A | F: TGCGAGTGGATGTATGA R: CCCACCTTACAGCAATATC | (AG) | 60 | 175–193 | 9 | FAM |
| CE2–5D | F: AGACGTAAGGTTTGAAGAGA R: ACAACTATGCCAAATTAAGTAT | (AC) | 60 | 202–206 | 3 | HEX |
| CE2–12D | F: TCCGCTAAATTATCATGG R: TCGAGTGCATAAAGGAATA | (AG) | 60 | 127–143 | 8 | FAM |
| CE2–4E | F: ATACAAAAGAATATGAAGTAATACA R: GTGTGCTTATGTATCTGGTAT | (AC) | 50 | 135–179 | 19 | HEX |
| Card 8 | F:TCGCCGTCTATTCTGTCGTTA  R: CTATTATCGGCAATGTGC | (AC) | 54 | 118–132 | 5 | FAM |
| Cobs 13 | F:TATCTTTTCAACCCTCTCGC R: TATTCCGCGATAGCTTAAAT | (CT) | 60 | 74-86 | 5 | TET |
